# ECE-VDTDA: A robust and computationally efficient collision avoidance system for driver assistance in foggy weather

**DOI:** 10.1371/journal.pone.0342186

**Published:** 2026-02-12

**Authors:** Naeem Raza, Muhammad Asif Habib, Abdullah M. Albarrak, Mudassar Ahmad, Alaa Eldeen Sayed Ahmed, Muhammad Yasir, Habib Ur Rahman, Muhammad Ahsan Latif

**Affiliations:** 1 Department of Computer Science, National University of Modern Languages, Islamabad, Faisalabad Campus, Faisalabad, Punjab, Pakistan; 2 Department of Computer Science, National Textile University, Faisalabad, Punjab, Pakistan; 3 College of Computer and Information Sciences, Imam Mohammad Ibn Saud Islamic University (IMSIU), Riyadh, Saudi Arabia; 4 Department of Computer Science, University of Engineering and Technology Lahore, Faisalabad Campus, Faisalabad, Punjab, Pakistan; 5 Department of Software Engineering, The University of Lahore, Lahore, Punjab, Pakistan; 6 Department of Computer Science, University of Agriculture, Faisalabad, Punjab, Pakistan; Nanjing Forestry University, CHINA

## Abstract

Advanced Driver Assistance Systems (ADAS) and Collision Avoidance Systems (CAS) are the primary modules of modern human-centric and autonomous driving applications, such as forward and rear-end collision warnings. To enhance the performance of ADAS and CAS systems in foggy weather, an Efficient and Cost-Effective Vehicle Detection and Tracking with Driver Assistance (ECE-VDTDA) system is proposed. The proposed ECE-VDTDA system comprises vehicle detection, tracking, and driver assistance modules. An optimized SimYOLO-V5s_WIOU vehicle detection algorithm is proposed, based on the SimSPPF module, the baseline You Only Look Once (YOLO) algorithm (YOLO-V5s), and the Wise Intersection Over Union (WIOU) localization loss function. State-of-the-art Deep-SORT, Strong-SORT, and optimized Deep-SORT algorithms are utilized for vehicle tracking. The vehicle detection and tracking performance of the ECE-VDTDA system is rigorously evaluated on DAWN, foggy driving, foggy cityscapes, BDD100K, web-collected, and self-collected foggy weather datasets. Optimized SimYOLO-V5s_WIOU algorithm outperformed on the foggy driving dataset with a 17.45% increase in mAP50, and foggy cityscapes dataset with a 0.32%, 1.05%, 1.58%, 2%, 0.54% increase in the multiclass mAP50, mAP50-95, F1 score, precision, and recall scores, respectively, compared to the baseline YOLO-V5s. Furthermore, the SimYOLO-V5s_WIOU algorithm also outperformed the state-of-the-art methods and enables Deep-SORT, Strong-SORT, and optimized Deep-SORT vehicle tracking algorithms to track vehicles with high confidence. The driver assistance module of the ECE-VDTDA system helps prevent imminent road collisions in foggy weather by estimating distance, speed, and time-to-collision and by issuing timely collision warnings. The experimental results demonstrate the robustness and computational efficiency of the proposed ECE-VDTDA system.

## 1 Introduction

Road traffic crashes cause a significant increase in global fatalities, traffic congestion, and property damage, necessitating the development, testing, and real-world implementation of efficient and cost-effective solutions for driver assistance. Vehicle detection enables a plethora of use cases, including vehicle tracking, counting, speed and distance estimations, traffic surveillance, traffic signs, anomaly detection, intelligent transportation systems, collision detection, and avoidance [1].

- Road traffic crashes can be effectively minimized by utilizing Advanced Driver Assistance Systems (ADAS) and Collision Avoidance Systems (CAS) with high-accuracy algorithms. Today, available ADAS and CAS systems have several limitations in terms of crash use-cases, false and missed detections, and are less adaptable to complex and specifically foggy weather conditions. Real-time and robust road perception and prediction techniques can only help ADAS systems to be proactively and timely aware of potential road collisions, and generate appropriate collision warnings to avoid them efficiently [2].
- Traditional CAS and ADAS systems are primarily based on ultrasonic or LiDAR sensors, have limitations in detection range, accuracy, and cost [3,4]. Some advanced CAS systems integrate diverse sensors, such as cameras, Light Detection and Ranging (LiDAR), Radio Detection and Ranging (RADAR), and Vehicle-to-Everything (V2X) communication technologies, along with machine learning algorithms, to autonomously detect and respond to potential road hazards, thereby avoiding collisions. These systems face challenges, including sensor noise, low accuracy in adverse weather conditions, communication delays, and security risks. Significant improvements in detection accuracy of sensors, support of edge computing technologies, and decision-making algorithms are still required for safer and autonomous driving [5].
- Vehicle detection and tracking are key components for vision-based speed and distance estimation methods, as they enable the detection and tracking of vehicle positions in frames, which requires camera calibration. Efficient vehicle speed estimation helps enforce speed limits in ITS and CAS systems [6]. You Only Look Once (YOLO) algorithm is widely used for vision-based vehicle speed and distance estimations. In a study, nano, small, and medium models of YOLO-V8 algorithm and Region of Interest (ROI) were utilized for precise and efficient bidirectional lane detection. The speed estimation is based on image or video frame coordinates [7].
- The CAS systems based on warnings can be classified into two categories: Forward Collision Warning (FCW) and Rear-end Collision Warning (RCW) systems [8]. Generally, collision avoidance involves two main steps: path planning and path tracking. In the path planning phase, collision-free path trajectories are generated, and in the path tracking phase, the generated collision-free path is accurately followed [9]. The process of decision-making for accurate trajectory generation, prediction, and path planning is essential for safely navigating roads and avoiding collisions in autonomous vehicles. The static and dynamic object detection, directional information, positions, velocities, and time headway are the basic parameters for future path planning and trajectory generation.

**Fig 1:**
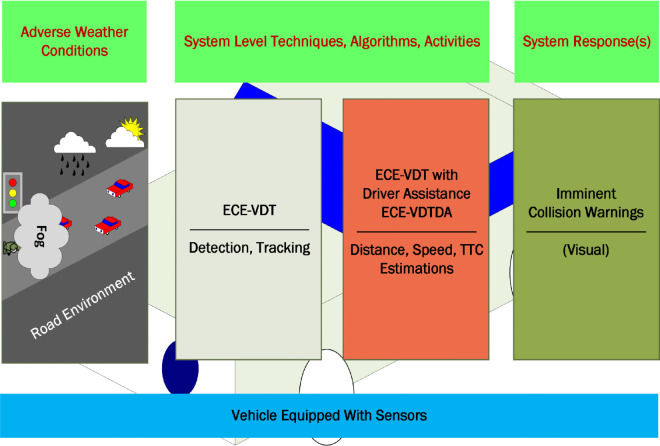
Abstract-level overview of ECE-VDTDA system.

**Fig 2:**
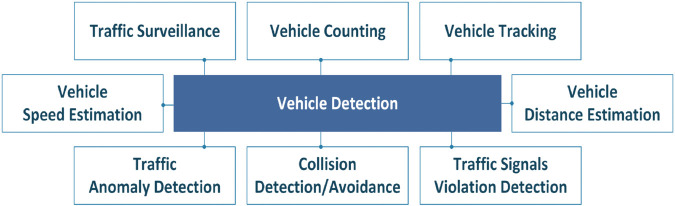
Applications of vehicle detection.

The generation of trajectories through deep-learning algorithms requires data from either cost-effective cameras or high-cost LiDAR sensors. The robust path planning through the Potential Field (PF) algorithm and trajectories generation is affected by the misinterpretation of the object’s occlusion and intension, sensor noise, overfitting, and errors in data collection methods [10–12]. Surrogate Safety Measures (SSMs) are the state-of-the-art safety indicators used for proactively assessing the road safety and potential crash risks in ADAS and CAS systems. Several SSM indicators based on temporal positions of the conflicting vehicles are Time-To-Collision (TTC), time exposed TTC, time integrated TTC, modified TTC, post-encroachment time, time to accident, anticipated collision time, headway, and crash index [13–15]. A collision avoidance scenario can be handled by braking and by steering [16]. The vehicle driving scenarios are also classified as autonomous driving, cooperative driving, and autonomous and cooperative driving [17–19]. The detailed design, functioning of driving scenarios, application specific use-cases of driver assistance, and collision avoidance scenarios are summarized in Table 1, and an abstract-level overview of the proposed Efficient and Cost-Effective Vehicle Detection and Collision Avoidance with Driver Assistance (ECE-VDTDA) system is illustrated in Fig 1, and vehicle detection use-cases are highlighted in Fig 2.

**Table 1:** State-of-the-art vehicle driving scenarios [17–19].

Driving and Application Use-cases	Description
Autonomous Driving (Partial, Semi, or Fully)	Autonomous driving vehicles are equipped with Advanced Driver Assistance Systems (ADAS), which include on-board sensors, cameras, RADAR, and LiDAR to detect obstacles on the road. The system assists human-centric drivers and autonomous vehicles in maintaining a safe distance and speed while also providing various warnings and alerts.
Cooperative Driving	Cooperative driving vehicles are equipped with V2V, V2P, V2I, V2N, and V2X communication technologies, enabling the exchange of information with nearby vehicles and infrastructure. This includes data on road and driving conditions such as traffic congestion, road blockages, construction zones, road surface damage, nearby fuel stations, as well as longitudinal and lateral driving behavior, along with alerts and warnings.
Autonomous and Cooperative Driving	Autonomous and cooperative driving vehicles leverage the combined capabilities of ADAS and V2X communication technologies to enable information-rich, reliable, and safer driving. By integrating on-board sensors, cameras, RADAR, and LiDAR for obstacle detection, and enabling real-time communication between vehicles, infrastructure, pedestrians, and networks, they support cooperative driving through the sharing of critical information, such as traffic congestion, road hazards, construction zones, fuel station availability, and driving behavior alerts.
Application Use-cases	Adaptive Cruise Control (ACC), Autonomous Emergency Breaking (AEB), Autonomous Emergency Steering (AES), Forward Collision Warning (FCW), Rear Collision Warning (RCW), Lane Departure Warning(LDW), Lane Keeping Assist (LKA), Lane Centering Assist (LCA), Curve Speed Warning (CSW), Surround View (SV), Blind Spot Warning (BSW), Assisted Parking (AP), Traffic Sign Recognition (TSR), Traffic Jam Assist (TJA), Driver Monitoring System (DMS)

**The main contributions of the presented research work are:**

- **Robust and Computationally Efficient ECE-VDTDA System:** The Efficient and Cost-Effective Vehicle Detection and Tracking (ECE-VDT) with Driver Assistance (ECE-VDTDA) system is proposed for collision avoidance and driver assistance in foggy weather.
- **SimYOLO-V5s_WIOU Vehicle Detection Algorithm:** The optimized SimYOLO-V5s_WIOU algorithm is proposed for vehicle detection based on Simplified Spatial Payramid Pooling Fast (SimSPPF) module, baseline YOLO-V5s [20,21] model, and Wise Intersection Over Union (WIOU) [22] localization loss function.
- **Vehicle Tracking Algorithm:** Baseline Deep-SORT [23,24], optimized Deep-SORT [25], and Strong-SORT [26,27] algorithms are utilized for vehicle tracking in the ECE-VDT module of the proposed ECE-VDTDA system.
- **Robust Performance Evaluation:** The vehicle detection performance is evaluated on diverse Foggy Driving (FD) [28,29], Vehicle Detection in Adverse Weather Nature (DAWN) [30,31], and Foggy Cityscape (FC) [28,32] foggy weather image datasets. The vehicle tracking performance is evaluated on diverse BDD100K datasets [33], web-collected [34], and self-collected [35] foggy weather video datasets.
- **Speed, Distance, and Time-To-Collision (TTC) Estimations:** Vision-based Vehicle Distance Estimation (VDE), Vehicle Speed Estimation (VSE), and Time-To-Collision (TTC) estimation methods are proposed. The bounding box height is considered the primary factor in these estimations.
- **Collision Alerts:** The VDE, VSE, and TTC estimations based on threshold levels enable the ECE-VDTDA system to generate collision alerts/warnings for enhanced driver assistance to avoid imminent road collisions in foggy weather.

The overall organization of the paper is as follows: The Introduction section discusses the importance of collision avoidance and driver assistance systems, the Literature review section presents the working of the You Only Look Once (YOLO) algorithm, as well as the State-Of-The-Art (SOTA) literature with related works highlighted. The Materials and methods section provides the design and implementation details of the proposed ECE-VDTDA system along with distance, speed, and TTC estimations. The Results and discussion section analyzes and compares the performance of the optimized SimYOLO-V5s_WIOU vehicle detection algorithm, optimized Deep-SORT and Strong-SORT SOTA vehicle tracking algorithms, VDE, VSE, and TTC estimations, and collision alerts. Finally, the Conclusion and future work section concludes the overall contribution of the proposed ECE-VDTDA system and provides directions for future work.

## Literature review

The state-of-the-art, single-stage, deep learning, and Convolutional Neural Network (CNN) architecture, You Only Look Once (YOLO) algorithm, consists of convolutional layers to form a Fully Convolutional Neural Network (FCN) architecture. Initially, 24 convolutional layers with two fully connected layers were used in the design of YOLO Version 1 (YOLO-V1). A super-fast, Fast YOLO also proposed, comprises nine convolutional layers. YOLO is invariant to the image size. The YOLO algorithm only looks once at the whole image and provides predictions. Fundamentally, the YOLO algorithm divides the input image into S*S sized grid cells. Each grid cell is subject to 5 predictions (bx,by,bw,bh,confidence score) based on B bounding boxes. Where *bx* and *by* are the coordinates of the center of the bounding box, whereas *bw* represents the width and *bh* represents the height of the bounding box. Mathematically, the bounding box elements are expressed in Eqs (1) and (2). The confidence score is associated with each bounding box. It also provides *C* class probabilities for the object classes being detected. For the on-road scenario, object classes can be vehicles, pedestrians, road signs, traffic lights, etc. The predictions of the YOLO are encoded as a tensor of size S*S*(B*5+C) [36].

$$b_{x}=\frac{x_{\min}+x_{\max}}{2W},\;b_{y}=\frac{y_{\min}+y_{\max}}{2H}$$ (1)

$$b_{w}=\frac{x_{\max}-x_{\min}}{2W},\;b_{h}=\frac{y_{\max}-y_{\min}}{2H}$$ (2)

A vision-based, efficient, and reliable framework for the ADAS system is proposed, utilizing the YOLO algorithm for vehicle detection and collision avoidance. The system supports driver assistance in urban and autonomous driving conditions on highways. It also supports Ultra-Fast Lane Detection (UFLD) for maintaining a safe lane to avoid potential collision risks [4]. A vision-based FCW framework is proposed, utilizing the YOLO-V5s algorithm for vehicle detection and the Kalman Filter (KF) for vehicle tracking. The FCW framework also supports driver assistance by incorporating leading vehicle speed and distance estimation as well as threshold-based TTC warnings. The experiments are performed on the BDD100K and DAWN datasets [37].

Attention Mechanism (AM) based variants, AMYOLO-V5s, were proposed for efficient and cost-effective vehicle detection in foggy weather. They evaluated the vehicle detection performance of AMYOLO-V5s variants on Google Colab and a local workstation system by utilizing the state-of-the-art Foggy Driving (FD) and Vehicle Detection in Adverse Weather Nature (DAWN) datasets [38]. Another updated study proposed an efficient and cost-effective SimYOLO-V5s varinst based on the SimSPPF module and diverse localization loss functions such as Complete Intersection Over Union (CIOU), Distance Intersection Over Union (DIOU), Efficient Intersection Over Union (EIOU), Generalized Intersection Over Union (GIOU), and SCYLLA Intersection Over Union (SIOU). They evaluated the vehicle detection performance of SimYOLO-V5s variants on Google Colab and a local workstation system by utilizing the state-of-the-art FD, DAWN, and Foggy Cityscapes (FC) datasets. They also utilized optimized Deep-SORT and Strong-SORT algorithms for vehicle tracking and evaluated the real-time performance on foggy video and the Burkey Deep Driven (BDD100K) datasets’ video sequences [25]. The vehicle detection performance of the YOLO-V11 algorithmic models was also evaluated on Google Colab by utilizing the FD and DAWN foggy datasets [39]. Another study focused on the social Vehicle-to-Everything (V2X) communication framework based on Software-Defined Networking (SDN) and 5G cellular infrastructure. Their framework highlighted the strength of Vehicle-To-Vehicle (V2V), Vehicle-To-Infrastructure (V2I), Vehicle-To-Network (V2N), and Vehicle-To-Padistrain (V2P) communication scenarios for effective driver assistance, traffic surveillance, and ITS [40]. A similar study considered a cellular and millimeter wave communication model for vehicular cloud computing [41]. The YOLO and Deep-SORT algorithms are widely used for detection and counting tasks [42–44]. A vision-based vehicle detection and speed estimation method was proposed by utilizing a monocular camera and the YOLO-V6 algorithm. The performance is primarily evaluated using the BrnoCompSpeed, focusing on detection accuracy in terms of recall, precision, and mAP scores, as well as speed in terms of FPS. Mean and median errors in detecting speed in Km/h are also presented [6]. Another study utilizes YOLO-V5s and Deep-SORT algorithms for speed estimation, employing a camera and RADAR for a multi-sensor methodology. They evaluated the performance on the vehicle re-identification dataset [45]. The distance estimation method based on the YOLO-V8 algorithm was proposed, and its performance was evaluated on the PASCAL VOC dataset [46]. Raspberry Pi and Radxa Zero enable a vision and deep-learning-based distance estimation method, which was proposed for cost-effective ADAS [47]. Another study proposed an object distance estimation method for ADAS using camera optics and image-based [48].

## Materials and methods

## The Efficient and Cost-Effective Vehicle Detection and Tracking with Driver Assistance (ECE-VDTDA) system

**Fig 3:**
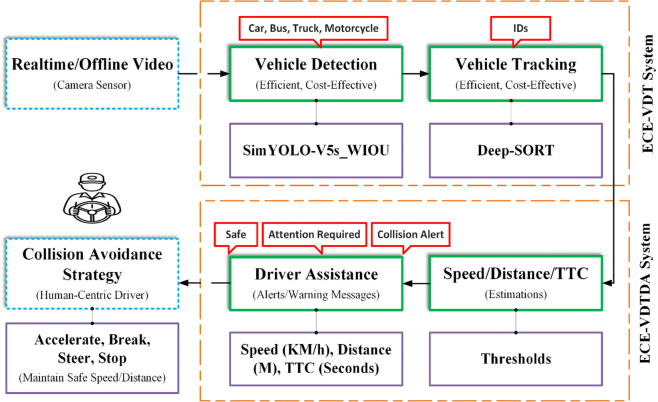
Research methodology of ECE-VDTDA system.

The Efficient and Cost-Effective Vehicle Detection and Tracking (ECE-VDT) with Driver Assistance (ECE-VDTDA) system is proposed, comprising ECE-VDT systems followed by a driver assistance module. Input images/video stream from the vehicle camera passes through the ECE-VDT system for effective and efficient vehicle detection and tracking. Vehicle detection is achieved through the designed and implemented SimYOLO-V5s_WIOU algorithm. The vehicle tracking is achieved through the optimized Deep-SORT algorithm. Using the tracking-by-detection methodology, the Deep-SORT algorithm employs an efficient and cost-effective SimYOLO-V5s_WIOU algorithm to track on-road vehicles. The ECE-VDT system is implemented and evaluated on DAWN, FD, and FC datasets, and tracking is implemented and evaluated on foggy videos. The ECE-VDT system provides vehicle detection and tracking information for four types of vehicles, including cars, buses, trucks, and motorcycles. The driver assistance module is designed to provide the driver with warnings of imminent collisions. The driver is responsible for taking preventive measures to avoid collisions with on-road vehicles. The collision warning or alert levels can be modeled as safe, warning, braking, steering, and pre-crash for human-centric and autonomous vehicles. The ECE-VDTDA system and components are illustrated in Fig 3.

### ECE-VDTDA system: Vehicle detection

**Fig 4:**
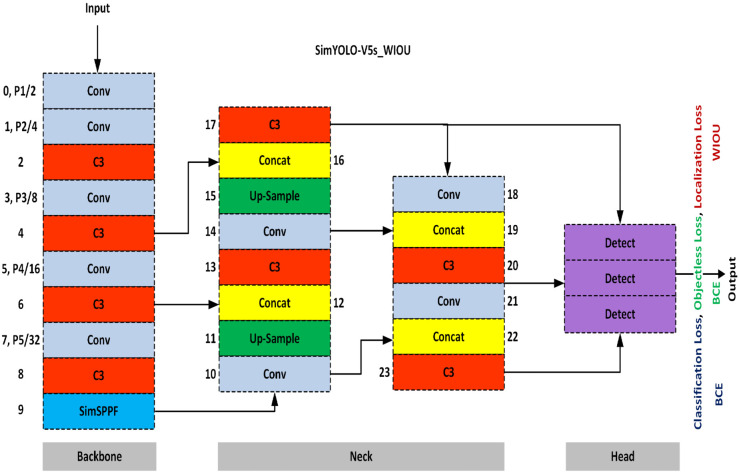
Architectural diagram of SimYOLO-V5s_WIOU vehicle detection algorithm.

To achieve high inference speed with competitive detection accuracy, the state-of-the-art You Only Look Once (YOLO) algorithm is selected to optimize for efficient and cost-effective vehicle detection in foggy weather. The state-of-the-art small model of the YOLO Version 5 (YOLO-V5s) algorithm is utilized as the base model for evaluating vehicle detection performance. The baseline YOLO-V5s model is optimized by introducing the Simplified Spatial Pyramid Pooling Fast (SimSPPF) module in the backbone network, and Wise Intersection Over Union (WIOU) localization loss function [22] is utilized for enhanced Bounding Box Regression (BBR) by the detection head in the proposed optimized SimYOLO-V5s_WIOU variant. The original SimYOLO-V5s algorithm and its variant were proposed in the latest and previous work [25]. Five variants of SimYOLO-V5s were proposed in previous work, based on five diverse localization loss functions: Complete Intersection Over Union (CIOU), Distance Intersection Over Union (DIOU), Efficient Intersection Over Union (EIOU), Generalized Intersection Over Union (GIOU), and SCYLLA Intersection Over Union (SIOU). The variants achieved state-of-the-art performance in both speed and accuracy for detecting vehicles in foggy weather. The variants were termed as SimYOLO-V5s_CIOU, SimYOLO-V5s_DIOU, SimYOLO-V5s_EIOU, SimYOLO-V5s_GIOU, and SimYOLO-V5s_SIOU. In this research work, SimYOLO-V5s_WIOU variant of the state-of-the-art SimYOLO-V5s algorithm is proposed in this research work. The architectural diagram of the optimized SimYOLO-V5s_WIOU algorithm and modules is illustrated in Fig 4. SimYOLO-V5s_WIOU algorithm consists of a feature extraction backbone, a feature fusion and aggregation neck, and a final detection head. The backbone consists of convolutional layers, Cross-Stage Partial (CSP) bottleneck C3 layers, and Simplified Spatial Pyramid Pooling (SimSPPF) module. The feature fusion and aggregation neck consists of the Path Aggregation Network (PANET), comprising convolutional layers, Cross-Stage Partial (CSP) bottleneck C3 layers, up-sampling, and concatenation connections. The final detection head performs detection on three scales to handle detections for small, medium, and large objects. The detection head performs detections based on objectness, classification, and localization scores, as well as loss functions based on them. The SimYOLO-V5s_WIOU algorithm for vehicle detection is based on diverse anchor boxes. The details of these anchor boxes based on image width, height, area, aspect ratio, and map size are summarized in Table 2.

**Table 2:** SimYOLO-V5s_WIOU anchor boxes grouped by detection layer, stride, and object size (input size 640×640).

Layer (stride, size)	Anchor #	Width (px)	Height (px)	Area (px^2^)	Aspect (w:h)	Map size
P3 (8, Small)	1	10	13	130	0.77:1	1.25 × 1.63
	2	16	30	480	0.53:1	2.00 × 3.75
	3	33	23	759	1.43:1	4.13 × 2.88
P4 (16, Medium)	4	30	61	1,830	0.49:1	1.88 × 3.81
	5	62	45	2,790	1.38:1	3.88 × 2.81
	6	59	119	7,021	0.50:1	3.69 × 7.44
P5 (32, Large)	7	116	90	10,440	1.29:1	3.63 × 2.81
	8	156	198	30,888	0.79:1	4.88 × 6.19
	9	373	326	121,598	1.14:1	11.66 × 10.19
**AutoAnchor Coverage with Anchors per target:** 5.46 (DAWN), 4.87 (FD), 4.79 (FC)						

#### IOU-Based loss functions.

**Fig 5:**
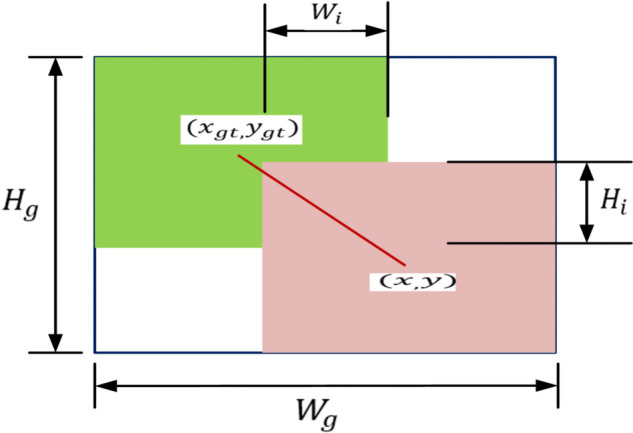
Bounding boxes and intersection over union with central points (Red) and smallest enclosing box (blue) [22].

Intersection Over Union (IOU) is the classical Bounding Box Regression (BBR) localization loss function that measures the overlap between the anchor-box and the Ground Truth (GT) box. The relationship of overlap between the anchor-box and the Ground Truth (GT) is illustrated in Fig 5. IOU loss function attempts to balance the learning of small and large objects for enhanced object detection [49]. Mathematically, the IOU loss functions (IOU, DIOU, CIOU, EIOU, SIOU) are expressed in Eqs (3–18).

$${ℒ}_{\text{IoU}}=1-\text{IoU}=1-\frac{W_{i}H_{i}}{S_{u}}$$ (3)

$$S_{u}=wh+w_{\text{gt}}h_{\text{gt}}-W_{i}H_{i}$$ (4)

$${ℒ}_{i}={ℒ}_{\text{IoU}}+R_{i}$$ (5)

The Distance Intersection Over Union (DIOU) loss function [50] performed BBR based on the normalized distance of the central points of the bounding boxes. Mathematically, the DIOU loss function is defined as

$$R_{\text{DIoU}}=\frac{(x-x_{\text{gt}}{)}^{2}+(y-y_{\text{gt}}{)}^{2}}{W_{g}^{2}+H_{g}^{2}}$$ (6)

The Complete Intersection Over Union (CIOU) loss function [50] enhances the DIOU loss function by incorporating the aspect ratio of bounding boxes. Mathematically, the CIOU loss function is defined as

$$R_{\text{CIoU}}=R_{\text{DIoU}}+\alpha v$$ (7)

$$\alpha =\frac{v}{{ℒ}_{\text{IoU}}+v}$$ (8)

Where *v* represents the aspect ratio.

$$v=\frac{4}{\pi^{2}}{\left(\tan^{-1}\left(\frac{w}{h}\right)-\tan^{-1}\left(\frac{w_{\text{gt}}}{h_{\text{gt}}}\right)\right)}^{2}$$ (9)

The Efficient Intersection Over Union (EIOU) loss function [51] is the enhanced version of the DIOU loss function. In EIOU, the penalty for violating the distance metric is increased. Mathematically, the EIOU loss function is defined as

$$R_{\text{EIoU}}=R_{\text{DIoU}}+\frac{(w-w_{\text{gt}}{)}^{2}}{W_{g}^{2}}+\frac{(h-h_{\text{gt}}{)}^{2}}{H_{g}^{2}}$$ (10)

The SCYLLA Intersection Over Union (SIOU) loss functions [52] consider the angle cost, distance cost, and shape cost for BBR. Mathematically, the SIOU loss function is defined as

$$R_{\text{SIoU}}=\Delta +\Omega$$ (11)

$$\Lambda =\sin\left(2\sin^{-1}\left(\frac{\min\left(|x-x_{\text{gt}}|,|y-y_{\text{gt}}|\right)}{\sqrt{(x-x_{\text{gt}}{)}^{2}+(y-y_{\text{gt}}{)}^{2}}+\varepsilon }\right)\right)$$ (12)

Where Λ introduces the angle cost. Λ = 0, when center points are aligned to the *x*–*axis* or *y*–*axis*, and Λ = 0, when aligned to 45 degrees to the *x*–*axis*.

$$\Delta =\frac{1}{2}\sum_{t\in \{w,h\}}\left(1-e^{-\gamma \rho_{t}}\right),\;\gamma =2-\Lambda$$ (13)

Where *Δ* introduces the distance cost.

$$\rho_{x}={\left(\frac{x-x_{\text{gt}}}{W_{g}}\right)}^{2}$$ (14)

$$\rho_{y}={\left(\frac{y-y_{\text{gt}}}{H_{g}}\right)}^{2}$$ (15)

$$\Omega =\frac{1}{2}\sum_{t\in \{w,h\}}{\left(1-e^{-\omega_{t}}\right)}^{\theta },\;\theta =4$$ (16)

Where Ω introduces the distance cost.

$$\omega_{w}=\frac{\left|w-w_{\text{gt}}\right|}{\max(w,w_{\text{gt}})}$$ (17)

$$\omega_{h}=\frac{\left|h-h_{\text{gt}}\right|}{\max(h,h_{\text{gt}})}$$ (18)

#### WIOU loss functions.

The Wise Intersection Over Union (WIOU) loss function [22], based on the standard IOU loss function and a dynamic non-monotonic Focusing Mechanism (FM), was proposed for BBR/localization. The dynamic non-monotonic FM primarily focuses on the outlier degree to effectively allocate gradients and improve the quality of anchor boxes, thereby enhancing the generalization performance of the object detection model. WIOU focuses on ordinary-quality anchor boxes to maintain a balance between high-quality and low-quality anchor boxes, thereby improving localization and detection performance. The mathematical expressions for the WIOU loss function and variants are provided in Eqs (19–25). The balance is achieved by assigning the small gradient gain to high-quality anchor boxes with small FM function *β* and the small gradient gain to low-quality anchor boxes with large FM function *β*.

$$\beta =\frac{{ℒ}_{\text{IoU}}}{{\overset{―}{ℒ}}_{\text{IoU}}}$$ (19)

Where *β* represents the outlier degree. A small outlier degree *β* corresponds to the high-quality anchor boxes. Three variants of the WIOU loss function for BBR were proposed as V1, V2, and V3.

WIOU Version 1 (WIOU-V1) is based on a distance attention mechanism. Mathematically, the WIOU-V1 loss function is defined as

$${ℒ}_{\text{WIoU-V1}}=R_{\text{WIoU}}\cdot {ℒ}_{\text{IoU}}$$ (20)

$$R_{\text{WIoU}}=\exp\left(\frac{(x-x_{\text{gt}}{)}^{2}+(y-y_{\text{gt}}{)}^{2}}{(W_{g}^{2}+H_{g}^{2}{)}^{*}}\right)$$ (21)

WIOU Version 2 (WIOU-V2) is based on monotonic FM. Mathematically, the WIOU-V2 loss function is defined as

$${ℒ}_{\text{WIoU-V2}}={ℒ}_{\text{IoU}}^{\gamma *}\cdot {ℒ}_{\text{WIoU-V1}},\;\gamma >0$$ (22)

The gradient gain *r* is defined as $r=L_{\text{IoU}}^{\gamma *}\in [0,1]$. Or equivalently,

$${ℒ}_{\text{WIoU-V2}}={\left(\frac{{ℒ}_{\text{IoU}}^{*}}{{\overset{―}{ℒ}}_{\text{IoU}}}\right)}^{\gamma }\cdot {ℒ}_{\text{WIoU-V1}}$$ (23)

Where ${\overset{―}{ℒ}}_{\text{IoU}}$ represents the exponential running average with momentum *m*.

WIOU Version 3 (WIOU-V3) is based on dynamic non-monotonic FM. Mathematically, the WIOU-V3 loss function is defined as

$${ℒ}_{\text{WIoU-V3}}=r\cdot {ℒ}_{\text{WIoU-V1}},\;r=\frac{\beta }{\delta \alpha^{\beta -\delta }}$$ (24)

Where *β* represents the outlier degree, r is the gradient gain, *α* and *β* are the hyperparameters.

$$\beta =\frac{{ℒ}_{\text{IoU}}^{*}}{{\overset{―}{ℒ}}_{\text{IoU}}},\;\beta \in [0,+\infty )$$ (25)

The SimSPPF module and SimYOLO-V5s_WIOU algorithm working in an algorithmic way are presented in Algorithms 1 and 2, respectively.

**Algorithm 1 SimSPPF.**

**Require:** Input feature map $X\in {\mathbb{R}}^{C\times H\times W}$

**Ensure:** Enriched multi-scale output feature map *O*

1: **function** SimConv(X,k,s,p)

2:   Apply 2D convolution on *X* with kernel size *k*, stride *s*, padding *p*

3:   Apply batch normalization

4:   Apply SiLU activation

5:   **return** activated feature map *Y*

6: **end function**

7: **Step 1: Channel Reduction**

8: $X_{1}\leftarrow \text{SimConv}(X,k=1,s=1,p=0)$     ▷ Reduce channels to *C*/2

9: **Step 2: Multi-Scale Pooling**

10: $Y_{1}\leftarrow \text{MaxPool}(X_{1},k=5,s=1,p=2)$

11: $Y_{2}\leftarrow \text{MaxPool}(Y_{1},k=5,s=1,p=2)$

12: $Y_{3}\leftarrow \text{MaxPool}(Y_{2},k=5,s=1,p=2)$

13: **Step 3: Feature Fusion**

14: Concatenate $Z=[X_{1},Y_{1},Y_{2},Y_{3}]$

15: O←SimConv(Z,k=1,s=1,p=0)     ▷ Fuse to *C* channels

16: **return** *O*

**Algorithm 2 SimYOLO-V5s_WIOU.**

**Require:** Input image $I\in {\mathbb{R}}^{H\times W\times 3}$

**Ensure:** Set of detection boxes *B*, scores *S*, classes *C*

1: **Input Preprocessing:**

    $I_{\text{resized}}\leftarrow \text{Resize}(I,640\times 640)$

    $I_{\text{norm}}\leftarrow \text{Normalize}(I_{\text{resized}})$

2: **Feature Extraction (Backbone - CSPDarknet):**

    $F_{3},F_{4},F_{5}\leftarrow \text{Backbone}(I_{\text{norm}})$

3: **Feature Aggregation (Neck - SimSPPF + PANet):**

    $F_{\text{fused}}\leftarrow \text{Neck}(F_{3},F_{4},F_{5})$

4: **Prediction Head:**

    $P\leftarrow \text{Head}(F_{\text{fused}})$

      • Bounding box coordinates (localization)

      • Objectness score (object presence)

      • Class probabilities (classification)

5: **Loss Computation:**

    **Localization Loss:** ${ℒ}_{\text{loc}}=\text{Wise-IoU}(B_{\text{pred}},B_{\text{gt}})$

    **Objectness Loss:** ${ℒ}_{\text{obj}}=\text{BCE}(s_{\text{pred}},s_{\text{gt}})$

    **Classification Loss:** ${ℒ}_{\text{cls}}=\text{BCE}(p_{\text{pred}},p_{\text{gt}})$

6: **Post-processing:**

    [B,S,C]←Decode(P)

    $\left[B^{\prime },S^{\prime },C^{\prime }]\leftarrow \text{NMS}(B,S,C\right)$

7: **return** $\left[B^{\prime },S^{\prime },C^{\prime }\right]$

### ECE-VDTDA system: Vehicle tracking

Simple Online and Realtime Tracking (SORT) [53] is a state-of-the-art, online, simple, and effective algorithm for the high accuracy and high precision Multi-Object Tracking (MOT) at higher Frame Per Second (FPS) rates. In the SORT algorithm, the Kalman Filter (KF) performs the task of State estimation and prediction of the objects in the image space. The Hungarian algorithm performs frame-by-frame data association with the help of an association metric to measure the overlap of bounding boxes. The higher number of Identity Switch (ID) and the miss detection/tracking of objects in occlusions are the major drawbacks of the SORT algorithm. To overcome the challenges of the SORT algorithm, the state-of-the-art Deep-SORT [23] algorithm utilizes a more informed deep association metric by combining the object’s appearance and motion features. The Deep-SORT algorithm is a state-of-the-art, real-time, online, efficient, and easy-to-implement algorithm. The Deep-SORT algorithm also utilizes an offline, pre-trained Convolutional Neural Network (CNN) on a large person re-identification dataset. The CNN network enhances the robustness of the Deep-SORT algorithm to address the challenges of a higher number of IDs and object occlusions over a longer period. Deep-SORT offers measurements to track associations in image space by utilizing nearest neighbor queries. The working of the state-of-the-art Deep-SORT algorithm for Multiple Object Tracking (MOT) in an algorithmic way is presented in Algorithm 3. State vector in the Deep-SORT at time *t* is defined in Eq (26):

$${𝐱}_{t}=[\begin{array}{cccccccc} u_{t} & v_{t} & \gamma_{t} & h_{t} & {\dot{u}}_{t} & {\dot{v}}_{t} & {\dot{\gamma }}_{t} & {\dot{h}}_{t} \end{array}{]}^{T}\in {\mathbb{R}}^{8}$$ (26)

Where:

- (u,v): center of the bounding box in image coordinates
- $\gamma =\frac{w}{h}$: aspect ratio
- *h*: height of the bounding box
- $\dot{u},\dot{v},\dot{\gamma },\dot{h}$: velocities of respective state components

**Algorithm 3 Deep-SORT: Kalman filtering for MOT.**

1: Initialize track set 𝒯←∅

2: **for** each frame *t* **do**

3:   **for** each track k∈𝒯 **do**

4:    Predict state ${𝐱}_{k}^{\left(t\right)}$ using Kalman filter

5:    Increment age: $a_{k}\leftarrow a_{k}+1$

6:   **end for**

7:   Obtain detections ${𝒟}^{\left(t\right)}=\{{𝐳}_{j}{\}}_{j=1}^{M}$

8:   Compute cost matrix *C*_*ij*_ using motion + appearance

9:   Perform assignment: associate tracks with detections

10:   **for** each matched pair (k,j) **do**

11:   Update Kalman filter with detection ${𝐳}_{j}$

12:   Reset age $a_{k}\leftarrow 0$

13:   **end for**

14:   **for** each unmatched track *k* **do**

15:    **if** $a_{k}>A_{\max}$ **then**

16:     Remove track *k* from 𝒯

17:    **end if**

18:   **end for**

19:   **for** each unmatched detection *j* **do**

20:    Initialize new track *k* with tentative status

21:    Add *k* to 𝒯

22:   **end for**

23:   **for** each tentative track *k* **do**

24:    **if** associated for $n_{\text{init}}$ consecutive frames **then**

25:     Promote to confirmed track

26:    **else if** not associated within $n_{\text{init}}$ frames **then**

27:     Delete track *k*

28:    **end if**

29:   **end for**

30: **end for**

The motion information metric is based on Mahalanobis distance using the *i*–*th* tracks measurement space $\left(y_{i},S_{i}\right)$ and the *j*–*th* bounding box detection *d*_*j*_.

$$d^{\left(1\right)}(i,j)=(d_{j}-y_{i}{)}^{T}S_{i}^{-1}(d_{j}-y_{i})$$ (27)

The decision is handled using binary variable decision of 1 if *i*–*th* track to *j*–*th* detection association is admissible using the Mahalanobis threshold of *t*^(1)^ = 9.4877 with the given metric.

$$b_{i,j}^{\left(1\right)}=[d^{\left(1\right)}(i,j)\leq t^{\left(1\right)}]$$ (28)

The appearance information metric is handled by the use of the smallest cosine distance between *i*–*th* tracks and the *j*–*th* bounding box detection *d*_*j*_.

$$d^{\left(2\right)}(i,j)=min\{1-r_{j}^{T}r_{k}^{i})|r_{k}^{i})\epsilon R_{i}\}$$ (29)

The decision is handled using a binary variable decision of *i*–*th* track to *j*–*th* detection association if admissible.

$$b_{i,j}^{\left(2\right)}=[d^{\left(2\right)}(i,j)\leq t^{\left(2\right)}]$$ (30)

The motion and appearance information metrics are combined as

$$c_{i,j}=\lambda \;d^{\left(1\right)}(i,j)+(1-\lambda )\;d^{\left(2\right)}(i,j)$$ (31)

The final association admissibility is handed as

$$b_{i,j}=\prod_{m=1}^{2}b_{i,j}^{\left(m\right)}$$ (32)

**Fig 6:**
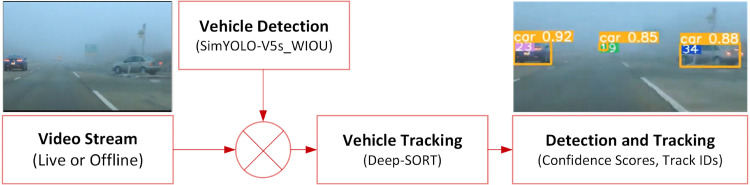
Vehicle detection and tracking in the ECE-VDTDA system.

The working of the ECE-VDT module of the ECE-VDTDA system is illustrated in Fig 6. The output of the ECE-VDT module shows the vehicle detection scores of the diverse vehicle classes on top of the bounding boxes, along with the tracking IDs. The output of the ECE-VDT module enables human-centric and autonomous vehicles to focus on a particular vehicle throughout the tracking period, facilitating imminent road collision detection and avoidance. The state-of-the-art Strong-SORT [26,27] algorithm, an improved version of the Deep-SORT algorithm, is also utilized to evaluate the vehicle tracking performance empowered by the robust and computationally efficient SimYOLO-V5s_WIOU vehicle detection algorithm in foggy weather. Strong-SORT enhances the Deep-SORT algorithm by introducing detection and embedding, along with inference boosting techniques. The key components of Deep-SORT are the YOLO-X model and BoT tracker with Exponential Moving Average (EMA), Correlation Coefficient maximization (ECC), vanilla Kalman filter, motion cost, and vanilla matching techniques. Strong-SORT utilizes an Appearance-Free Link model (AFLink) for enhancing the global association along with Gaussian-Smoothed Interpolation (GSI) for minimizing the missed detections.

### ECE-VDTDA system: Driver assistance and collision avoidance

**Fig 7:**
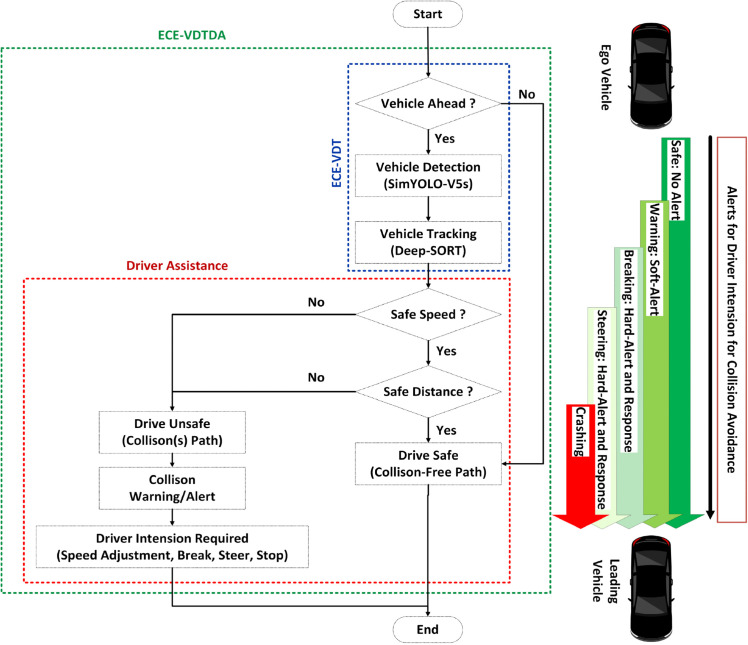
Flow chart of the ECE-VDTDA system.

Driver assistance is one of the essential areas of research in modern-day human-centric and autonomous vehicles. Vehicle drivers are the primary actors in human-centric vehicles, making decisions regarding steering, acceleration, navigation, and braking. The constant attention and interaction of human-centric vehicle drivers are required even with supportive driver assistance applications. Numerous ADAS applications introduced by the researchers for driver assistance include lane-keeping assistance, lane-departure warning, forward or rear-end collision warnings to avoid collisions, blind-spot warnings, cruise control, and safe parking, among others. Efficient and cost-effective ADAS systems primarily require single or multi-vehicle camera sensors. Depending on the application, camera sensors can operate as a single monocular camera, a binocular pair, or in a stereo arrangement for depth perception. The real-time vision-based vehicle detection and tracking assist drivers of human-centric vehicles in becoming aware of on-road vehicles for safe driving maneuvers. More specifically, forward collision warnings help drivers of human-centric vehicles to maintain a safe speed and distance from the leading vehicles on the road. The collision warnings can only be generated by utilizing the strength of a vision-based, efficient, and cost-effective vehicle detection and tracking system. The functioning of the designed and implemented ECE-VDTDA system depends on the input(s) from the designed and implemented ECE-VDT system. The more accurate and timely information from the ECE-VDT system will result in fewer false positives, generating more stable imminent collision warnings for drivers. The algorithmic working of the proposed ECE-VDTDA system is presented in Algorithm 4. The vehicle detection module in ECE-VDT can efficiently and cost-effectively detect the leading vehicles on the road, with confidence scores displayed above each vehicle. The vehicle tracking module uses the vehicle detection module predictions and confidence scores to track the leading vehicles. The classical Kalman filter algorithm for state estimation empowers the ECE-VDT vehicle tracking module in ECE-VDT. Hungarian algorithms for data associations assign track IDs to each leading vehicle. The assigned track IDs are displayed above each vehicle. The ECE-VDT system is primarily personalized for cars, buses, and trucks. As we know, highways and expressways only allow cars, buses, and trucks to be on the road. The integration of driver assistance functionality with ECE-VDT will further extend the system’s capabilities, warning human-centric drivers to avoid imminent road traffic collisions. The driver assistance system requires distance and speed estimations for the CAS system to generate alerts. The notifications or alerts can be based on visual, auditory, or tactile signals. The centrally focused CAS systems use case is forward collision warnings in this research work. The overall flow chart illustrates the overall working, and the modules are shown in Fig 7.

**Algorithm 4 ECE-VDTDA system (Collision alerts for collision avoidance and driver assistance).**

1: **Start**

2: **Phase-1: System Initialization**

3: Initialize(*camera_params*, *SimYOLO-V5s_WIOU model*, *Deep-SORT tracker*, *hyperparameters*, *ROI*, *logging*, *hardware*)

4: dict_info ←{}, alert_dict ←{}, initial_frame_info ←{}

5: **Phase-2: Vehicle Detection**

6: **for** *each frame* f∈video_stream **do**

7:   pred←SimYOLO−V5s_Inference(f)

8:   detections←NMS(pred,conf_thres,iou_thres)

9:   filtered←FilterROI(detections,ROI)

10: **end for**

11: **Phase-3: Vehicle Tracking**

12: tracking_output←Deep−SORT.Update(filtered)

13: **for** each vehicle v∈tracking_output **do**

14:   id,bbox,cls←v

15:   Update(dict_info, initial_frame_info, id)

16: **end for**

17: **Phase-4: Driver Assistance (Collision Avoidance)**

18: **for** each tracked *id* with lifetime ≥ 20 frames **do**

19:   px_disp←dict_info[id]

20:   depth_m←EstimateDepth(bbox,camera_params)

21:   speed_kmh←PixelDispToSpeed(px_disp,depth_m,FPS)

22:   distance_m←depth_m

23:   TTC←distance_m/(speed_kmh/3.6)

24:   alert←ClassifyAlert(speed_kmh,distance_m,TTC)

25:   alert_dict[id] ←alert

26: **end for**

27: **for** each tracked vehicle *o* in tracking_output **do**

28:   **if** v.id∈alert_dict **and** Center(*o*.*bbox*) ∈ROI **then**

29:    AnnotateFrame(v.bbox, alert_dict[v.id])

30:    TriggerAlert(alert_dict[v.id])

31:    **if** alert indicates *collision risk* **then**

32:     *Driver can Apply Safe_Braking()* or

33:     *Driver can Apply Safe_Steering()* or

34:     *Driver can Maintain Safe_Speed()* or

35:     *Driver can Maintain Safe_Distance()*

36:    **end if**

37:   **else**

38:    AnnotateFrame(v.bbox, “track id: v.id”)

39:   **end if**

40: **end for**

41: **Phase-5: Adaptive System Tuning**

42: *TuneDetectionThresholds(road_condition, weather, traffic_density)*

43: *UpdateTrackerParams(based on occlusions, ID-switches)*

44: *OptimizeModelParameters()*

45: **End.**

Several assumptions are considered for estimating the speed and distance of leading vehicles, including a flat road, constant acceleration, and a small distance between the leading vehicle and the ego vehicle.

#### Vehicle Speed Estimation (VSE).

In this research work, the vision-based leading Vehicle Speed Estimation (VSE) methodology is utilized. The VSE process operates in a continuous loop, processing each video frame by frame. The process starts with efficient and cost-effective vehicle detection followed by vehicle tracking. Vehicle detection is achieved by the optimized SimYOLO-V5s_WIOU algorithm, and vehicle tracking by the optimized Deep-SORT algorithm. The bounding boxes are scaled for better localization. The speed estimation (km/h) process is directly based on the bounding box height (pixels) for all vehicle detection and tracking classes. The relationship between bounding box height and speed estimation is mathematically expressed in Eq (33).

$${\text{Speed}}_{\text{km/h}}=50+5\times \left(\frac{100}{\text{bbox\_height}}\right)$$ (33)

This ensures that larger (closer) bounding boxes and smaller (farther) bounding boxes result in a relatively constant speed value. The collision warnings for driver assistance based on speed estimation are configured with threshold levels. The threshold levels are summarized in Table 3. The track IDs, along with speed and collision alert warnings, are displayed on the vehicles’ bounding boxes for better visualization. For a vehicle with a speed above 60 (km/h), an imminent collision alert is generated, for a speed in the range of 53 (km/h) and 60 (km/h), an attention required alert will be generated, and for a speed below 53 (km/h), a safe alert will be generated for effective driver assistance.

**Table 3:** Threshold levels and warnings/alerts for vehicle speed, distance, and TTC estimations in the ECE-VDTDA system.

Function	Threshold	Warning Messages	Color
**VSE**	Above 60 Km/h	Imminent Collision Alert	Red
	53-60 Km/h	Attention Required	Yellow
	0-52 Km/h	Safe	White
**VDE**	0-10 meters	Imminent Collision Alert	Red
	11-50 meters	Attention Required	Yellow
	Above 50 meters	Safe	White
**TTC**	0-2 seconds	Imminent Collision Alert	Red
	2.1-5 seconds	Attention Required	Yellow
	Above 5 seconds	Safe	White

#### Vehicle Distance Estimation (VDE).

In this research work, the vision-based leading Vehicle Distance Estimation (VDE) methodology is utilized. The VDE process operates in a continuous loop, processing each video frame by frame. The process starts with efficient and cost-effective vehicle detection followed by vehicle tracking. Vehicle detection is achieved by the optimized SimYOLO-V5s_WIOU algorithm, and vehicle tracking by the optimized Deep-SORT algorithm. The bounding boxes are scaled for better localization. The distance estimation (in meters) is inversely proportional to the bounding box height (in pixels) for all vehicle detection and tracking classes. The bigger bounding boxes correspond to the lower distance and meet the real-world settings of close vehicles, with a smaller distance in meters, to avoid imminent road collisions, and vice versa, with the help of a scaling factor *k*1. The relationship of bounding box height and scaling factor *k*1 with distance is mathematically expressed in Eq (34).

$${\text{Distance}}_{\text{meters}}=\text{round}\left(\frac{k1}{\max(\text{bbox\_height},1)}\right)$$ (34)

The collision warnings for driver assistance based on distance estimation are configured with threshold levels. The threshold levels are summarized in Table 3. The track IDs, along with distance and collision alert warnings, are displayed on the vehicles’ bounding boxes for better visualization. For a vehicle with a distance less than 10 meters, an imminent collision alert is generated, for a distance in the range of 11 meters and 50 meters, an attention required alert will be generated, and for a distance above 50 meters, a safe alert will be generated for effective driver assistance.

#### Time-To-Collision (TTC) estimation.

Time-To-Collision (TTC) widely recognized Surrogate Safety Measures (SSMs) indicator is utilized for the collision avoidance and driver assistance in the proposed ECE-VDTDA system. TTC assists both human drivers and autonomous vehicles in taking timely preventive actions by assessing collision risk. TTC is widely recognized, straightforward to compute, and extensively as a reliable early-warning indicator for imminent collisions. It indicates the remaining time before the ego and leading vehicle collide, based on their relative speed and distance. This study focuses on real-time vision-based forward collision risk estimation using continuous frame-by-frame distance and speed estimation, and TTC provides a direct, threshold-based measure of imminent collision risk without requiring additional assumptions. In the proposed system, TTC is estimated in seconds and displayed on the leading vehicle’s bounding boxes. The higher TTC enables human-centric vehicle drivers or autonomous vehicles to safely travel, perform longitudinal or lateral driving maneuvers, and adjust speed and distance profiles more effectively. Other SSM indicators, while valuable in broader traffic safety analyses, were not considered in this study because they either involve cumulative exposure (e.g., TET, TIT) or require complex multi-vehicle interaction modeling (e.g., PET, CI), which is beyond the real-time focus of the proposed system. Furthermore, this study focuses on real-time vision-based forward collision risk estimation using continuous frame-by-frame distance and speed estimation, and TTC provides a direct, threshold-based measure of imminent collision risk without requiring additional assumptions [13–15]. The proposed ECE-VDTDA systems collision avoidance and driver assistance module utilizes vision-based distance and speed estimations to compute the TTC. Eqs (35) and (36) present the TTC formulations derived from the estimated speed and distance profiles. Specifically, Eq (35) expresses the fundamental relationship of TTC as the ratio of distance to speed (more specifically relative speed), assuming that the vehicles continue to move at a constant speed. This formulation is applicable to common driving scenarios, including car-following, head-on, and rear-end approaches [54–56]. The TTC estimation based on distance and speed is mathematically expressed in Eq (35).

$${\text{TTC}}_{\text{seconds}}=\frac{\text{Distance}}{\text{Speed}}$$ (35)

Where, the speed is assumed with a fixed constant speed of 5*m*/*s* or (18km/h). Eq (35) represents a simplified TTC formulation that does not account for angular collisions. This is because angular collision detection and avoidance require not only the inter-vehicle distance and relative speed, but also detailed information such as vehicle velocities in different directions, trajectory patterns, and path predictions, along with detailed mathematical formulation that fall outside the scope of this study. By tying TTC directly to the calculated distance, the warning system’s thresholds for both metrics are automatically synchronized. This is a deliberate design choice to provide a stable, consistent warning output rather than relying on noisy, frame-to-frame speed calculations. The TTC estimation based on speed is mathematically expressed in Eq (36).

$${\text{TTC}}_{\text{seconds}}=\left(\frac{{\text{speed}}_{\text{m/s}}}{{\text{fixed\_distance}}_{\text{m}}}\right)\times k_{2}$$ (36)

Where the factor *k*2 is used to adjust the TTC estimation in seconds. The collision warnings for driver assistance based on TTC estimation are configured with threshold levels. Eq (36) is introduced as an approximation for scenarios where the ego vehicle approaches a reference distance threshold at a nearly constant speed. In this context, the fixed distance represents the predefined safety margin (e.g., minimum safe following distance), while *k*2 is a scaling factor that converts the speed and distance ratio into time units consistent with the system’s frame rate, measurement scale, and unit normalization. Eq (36) is used as a normalized and computationally lightweight metric by the proposed ECE-VDTDA system for early-warning decisions when the distance is fixed-threshold or when the system monitors how quickly the vehicle is approaching a critical boundary. The threshold levels are summarized in Table 3. The track IDs, along with distance and collision alert warnings, and speed and collision warnings, are displayed on the vehicles’ bounding boxes for better visualization. For a vehicle with a TTC less than 2 seconds, an imminent collision alert is generated; for a TTC in the range of 2 seconds and 10 seconds, an attention required alert will be generated; and for a TTC above 10 seconds, a safe alert will be generated for effective driver assistance.

#### Collision risk.

The Collision Risk is directly linked with the TTC estimation. The smaller the TTC time, the higher the collision risk and vice versa. Mathematically, the collision risk based on the TTC is expressed in Eq (37):

$$Risk_{collision}=\frac{1}{TTC}$$ (37)

Eq (37) is derived from [56,57] used with the ECE-VDTDA systems collision avoidance and driver assistance module as a deterministic surrogate risk indicator widely adopted in real-time ADAS applications to reflect the severity of an imminent collision, rather than as a formal probabilistic crash-risk estimate, which would require statistical frameworks such as extreme value theory. Collision risk continuously changes as the TTC changes, which in turn changes as per the leading vehicle speed or distance changes. The proposed ECE-VDTDA system offers an efficient and cost-effective method for predicting the leading vehicles on the road, thereby enhancing the accuracy of estimations. Collision risk requires human-centric driver intervention to take preventive measures and avoid imminent collisions.

### Experimental setup

A diverse set of experiments was performed to evaluate the efficiency and cost-effectiveness of the ECE-VDTDA system and modules. The experiments are performed on a cloud-based Google Colab system and a local workstation system equipped with a Graphical Processing Unit (GPU) and various computer vision libraries and packages. The NVIDIA Tesla T4 GPU with 15 GB of memory is used for the experiments on Google Colab, whereas the NVIDIA GeForce GTX 1050 Ti GPU with 4GB of dedicated memory is used for the experiments on the local workstation system. The 640 image size was used throughout the experiments. The training is performed for 300 epochs on Google Colab and 100 epochs on a local workstation system. The Stochastic Gradient Descent (SGD) optimization algorithm, with a batch size of 16 for training and 1 for validation, utilizing its default training, validation, detection, and tracking hyperparameters, is employed for the experiments with YOLO-V5s. Vehicle tracking is performed with Deep-SORT and Strong-SORT algorithms with default configuration parameters. The confidence threshold of 0.25, the NMS IOU threshold of 0.45, the maximum age of 30, NN_Budget of 100, and the maximum detections of 1000 are considered. The collision avoidance and driver assistance functions require vision-based speed, distance, and Time-To-Collision (TTC) estimations.

#### Datasets.

**Fig 8:**
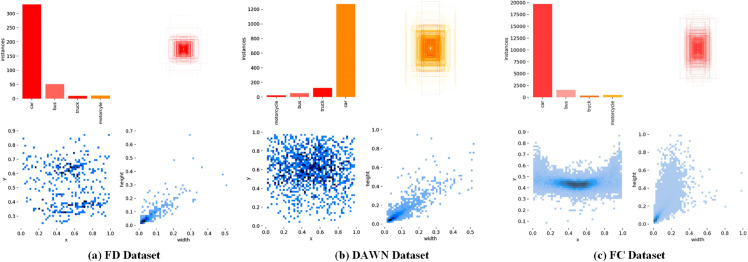
FD, DAWN, and FC datasets class-wise labels distribution.

The vehicle detection performance of the ECE-VDTDA system, specifically the optimized SimYOLO-V5s_WIOU, was evaluated on diverse foggy weather image datasets. The state-of-the-art Foggy Driving (FD) [28,29], Vehicle Detection in Adverse Weather Nature (DAWN) [30,31], and Foggy Cityscapes [29,32] image datasets are utilized. The FD dataset comprises 101 real foggy weather road vehicle detection images. These images were pre-processed, self-annotated, and customized using the YOLO Label [58] tool, and split into training and validation images with an 80:20 ratio. The DAWN dataset comprises 1000 diverse weather road vehicle detection images, including 300 foggy images. The foggy images were pre-processed, customized, and split into training and validation images with an 80:20 ratio. The FC dataset comprises 5000 synthetic foggy weather road vehicle detection images. These images were pre-processed, self-annotated, and customized using the YOLO Label tool, and split into training, validation, and test images with a 60:10:30 ratio. The visualization of the FD, DAWN, and FC datasets’ vehicle detection classes, instances, and labels distribution is illustrated in Fig 8.

The vehicle tracking performance of the ECE-VDTDA system, specifically the optimized Deep-SORT algorithm, as well as collision avoidance and driver assistance capabilities, was evaluated using diverse BDD100K datasets [33], web-collected [34], and self-collected [35] foggy weather video datasets. DAWN, FD, FC, BDD100K, and web-collected datasets used in this research are publicly available and were collected, processed, and analyzed in accordance with the terms and conditions specified by their original providers. In addition, the details of the self-collected [35] dataset have been included in the manuscript to support reproducibility and ensure transparency of the experimental setup. Furthermore, all publicly available datasets (DAWN, FD, FC, BDD100K, and web-collected) used in this study do not require permits. The self-collected [35] dataset was recorded in publicly accessible locations where no special permits or field-site approvals are required for non-invasive visual data collection.

#### Performance metrics.

A diverse set of state-of-the-art performance metrics was utilized to evaluate the efficiency and cost-effectiveness of the ECE-VDTDA system and modules.

**a) Precision (P):** Accuracy of correctly predicting true or positive objects over all objects predicted positive, including false positives, as expressed in Eq (38).

$$Precision=\frac{TP}{TP+FP}$$ (38)

**b) Recall (R):** Sensitivity of correctly predicting true or positive objects, overall predicted objects, including false negatives, as expressed in Eq (39).

$$Recall=\frac{TP}{TP+FN}$$ (39)

**c) Precision versus Recall Curve:** It maps the relationship between the precision and recall scores of an object detector, and its optimal point lies near (1.0, 1.0) of the precision-recall curve.

**d) F1 score:** Harmonic mean or a balanced measure of precision and recall. An enhanced F1 score for object detection requires higher precision and recall scores, as expressed in Eq (40).

$$F1Score=2\times \frac{Precision*Recall}{Precision+Recall}$$ (40)

**e) Average Precision (AP):** It is the area under the interpolated precision-recall curve, measuring the precision scores at various recall (r) scores. AP is mathematically expressed in Eq (41).

$$AP=1/11\sum_{r=0,0,1,0.2,....\mathrm{.1}}Pinterpolation(r)$$ (41)

**f) Mean Average Precision (mAP):** It is the mean value of the AP over N classes for multi-class object detection. Mainly, mAP50 is expressed at an IOU threshold of 50. mAP is mathematically expressed in Eq (42).

$$mAP=1/N\sum_{i=1}^{N}APi$$ (42)

**g) Frame Per Second (FPS):** It represents the number of frames processed per second to meet the real-time inference speed of the object detection algorithms. FPS is based on the overall processing time required by the algorithm. Higher FPS helps object detection algorithms to achieve high computational efficiency and real-time performance [59]. Mathematically, FPS is expressed as Eq (43):

$$FPS=\frac{1000}{Processing\;Time\;Per\;Frame\;(ms)}$$ (43)

**h) GPU Memory Utilization (GB):** It represents the total GPU memory the vehicle detection model utilizes during training. It depends on the model size, images, and batch size, etc.

**h) Training Time (Hours):** It represents the total training time required by the model to complete the training epochs. It depends on the model size, images, epochs, batch size, etc.

## Results and discussion

### ECE-VDTDA system: Vehicle detection

The proposed ECE-VDTDA system utilizes an optimized SimYOLO-V5s_WIOU algorithm for efficient and cost-effective vehicle detection in foggy weather conditions. The vehicle detection performance of the optimized SimYOLO-V5s_WIOU algorithm is evaluated using diverse metrics across various FD, DAWN, and FC foggy weather vehicle detection datasets.

#### SimYOLO-V5s_WIOU vehicle detection performance on the FD dataset.

The optimized SimYOLO-V5s_WIOU algorithm offers a multiclass vehicle detection mAP50 score of 47.3%, and a multiclass vehicle detection mAP50-95 score of 24% on the FD dataset. The SimYOLO-V5s_WIOU overall precision score is 26.7%, the recall score is 56.7%, and the balanced F1 score is 40%. SimYOLO-V5s_WIOU detects cars with a mAP50 score of 68.3%, buses with a mAP50 score of 37.4%, and trucks with a mAP50 score of 36.1%. When compared with the baseline YOLO-V5s algorithm, SimYOLO-V5s_WIOU outperforms as an effective and efficient vehicle detection algorithm in recall, multiclass mAP50, multiclass mAP50-95, and F1 scores, as well as detecting buses and trucks. Moreover, SimYOLO-V5s_WIOU also outperforms in pre-processing time, inference time, and post-processing time, and offers higher FPS compared to baseline YOLO-V5s. SimYOLO-V5s_WIOU requires less training time with moderately higher GPU memory requirements compared to baseline YOLO-V5s. The comparison of vehicle detection accuracy and speed results on the FD dataset are summarized in Table 4 and Table 5, respectively.

**Table 4:** Comparison of vehicle detection performance (accuracy) of baseline YOLO-V5s and SimYOLO-V5s_WIOU on the FD, DAWN, and FC datasets.

Model	Dataset	P	R	Car	Bus	Truck	Motorcycle	mAP50	mAP50-95	F1
Baseline YOLO-V5s [25]	FD	**35**	48.7	**71.6**	29.8	18.8	–	40.1	20.8	38
**SimYOLO-V5s_WIOU**	FD	26.7	**56.7**	68.3	**37.4**	**36.1**	–	**47.3**	**24**	**40**
Baseline YOLO-V5s [25]	DAWN	**82.5**	**68.4**	**88.1**	**35.7**	**77.4**	99.5	**75.2**	**41.1**	**72**
**SimYOLO-V5s_WIOU**	DAWN	77.5	67	87.2	30.8	76.2	**99.5**	73.4	39.4	68
Baseline YOLO-V5s [25]	FC	74.1	55.2	**82.5**	**63.8**	**61.1**	39.1	61.6	38	63
**SimYOLO-V5s_WIOU**	FC	**75.6**	**55.5**	82.3	62.9	58.3	**43.6**	**61.8**	**38.4**	**64**

**Table 5:** Comparison of vehicle detection performance (speed) of the baseline YOLO-V5s and SimYOLO-V5s_WIOU on the FD, DAWN, and FC datasets.

Model	Dataset	GPU-MU	Pre-Processing	Inference	Post-Processing	TT	FPS
Baseline YOLO-V5s [25]	FD	**3.67**	0.3	7.6	33.5	0.930	24
**SimYOLO-V5s_WIOU**	**FD**	**3.74**	**0.3**	**6.7**	**5.5**	**0.841**	**80**
Baseline YOLO-V5s [25]	DAWN	**4.19**	0.3	7.4	13.8	1.758	47
**SimYOLO-V5s_WIOU**	**DAWN**	**4.39**	**0.3**	**7.3**	**3.8**	**1.200**	**88**
Baseline YOLO-V5s [25]	FC	**3.5**	1.6	48.2	**2.1**	5.204	19
**SimYOLO-V5s_WIOU**	**FC**	**3.77**	**0.5**	**35.1**	**4.8**	**4.916**	**25**
GPU-MU: GPU Memory Utilization (GB); Pre-Processing, Inference, and Post-Processing (ms); TT: Training Time (Hrs)							

In comparison with State-Of-The-Art (SOTA) methods, the efficiency and cost-effectiveness of the optimized SimYOLO-V5s_WIOU algorithm is evident from the results summarized in Tables 6 and 7, which show that SimYOLO-V5s_WIOU outperforms in multiclass mAP50, mAP50-95, and F1 scores, and also detects trucks with a higher mAP50 score on the FD dataset. However, it also achieves competitive results when compared with the SOTA in recall, detecting cars and buses. Moreover, SimYOLO-V5s_WIOU also outperforms in pre-processing time, inference time, and post-processing time, and offers higher FPS compared to SOTA.

**Table 6:** Comparison of vehicle detection performance of SOTA and SimYOLO-V5s_WIOU on FD dataset.

Model	P	R	Car	Bus	Truck	mAP50	mAP50-95	F1 Score
YOLO-V11s [39]	**71.1**	37.8	67.4	**41.9**	17	42.1	21.5	37
YOLO-V5s+CBAM [38]	**30.5**	**63.2**	**70.8**	**37.9**	14.3	41	20.1	40
YOLO-V5s+NAM [38]	**72.5**	32.2	67.2	36.4	19.9	41.2	19.5	34
YOLO-V5s+SimAM [38]	**67.6**	30.6	**69.3**	33	12.5	38.2	19	32
**SimYOLO-V5s_WIOU**	26.7	56.7	68.3	37.4	**36.1**	**47.3**	**24**	**40**

**Table 7:** Comparison of vehicle detection performance (speed) of SimYOLO-V5s_WIOU with SOTA on the FD dataset.

Model	Pre-Processing (ms)	Inference (ms)	Post-Processing (ms)	FPS
YOLO-V11s [39]	1.8	30.1	55.1	12
YOLO-V5s+CBAM [38]	0.3	7.2	36.9	23
YOLO-V5s+NAM [38]	0.3	7.2	32.1	25
YOLO-V5s+SimAM [38]	0.3	6.8	34.6	24
**SimYOLO-V5s_WIOU**	**0.3**	**6.7**	**5.5**	**80**

#### SimYOLO-V5s_WIOU vehicle detection performance on the DAWN dataset.

The optimized SimYOLO-V5s_WIOU algorithm offers a multiclass vehicle detection mAP50 score of 73.4%, and a multiclass vehicle detection mAP50-95 score of 39.4% on the DAWN dataset. The SimYOLO-V5s_WIOU overall precision score is 77.5%, the recall score is 67%, and the balanced F1 score is 68%. SimYOLO-V5s_WIOU detects cars with a mAP50 score of 87.2%, buses with a mAP50 score of 30.8%, trucks with a mAP50 score of 76.2%, and motorcycles with a mAP50 score of 99.5%. When compared with the baseline YOLO-V5s algorithm, SimYOLO-V5s_WIOU shows competitive results and underperforms in precision, recall, mAP50, mAP50-95, and F1 scores. However, SimYOLO-V5s_WIOU outperforms in pre-processing time, inference time, and post-processing time, and offers higher FPS compared to baseline YOLO-V5s. SimYOLO-V5s_WIOU requires less training time with moderately higher GPU memory requirements compared to baseline YOLO-V5s. The comparison of vehicle detection accuracy and speed results on the DAWN dataset is summarized in Table 4 and Table 5, respectively.

In comparison to SOTA methods, the efficiency and cost-effectiveness of the optimized SimYOLO-V5s_WIOU algorithm is evident from the results of accuracy metrics as summarized in Table 8 on the DAWN dataset, which show that SimYOLO-V5s_WIOU outperforms in multiclass mAP50, mAP50-95, and precision scores, and also detects motorcycles and trucks with a higher mAP50 score on the DAWN dataset. However, it also achieves competitive results when compared with the SOTA in recall, F1 score, and detecting cars and buses. However, SimYOLO-V5s_WIOU also shows competitive results in terms of speed metrics, as summarized in Table 9, including pre-processing time, inference time, post-processing time, and FPS, compared to SOTA.

**Table 8:** Comparison of vehicle detection performance of state-of-the-art and SimYOLO-V5s_WIOU on DAWN dataset.

Model	P	R	Car	Bus	Truck	Motorcycle	mAP50	mAP50-95	F1
YOLO-V11s [39]	73.8	**69.1**	**88.2**	**36.3**	68.4	99.5	73.1	38.5	**71**
YOLO-V5s+CBAM [38]	47.7	**73.8**	86.7	25.1	73.5	99.5	71.2	35.8	61
YOLO-V5s+NAM [38]	77.1	58.5	86.4	**31.9**	71.9	49.8	60	30.8	65
YOLO-V5s+SimAM [38]	46.7	60.6	**87.6**	30.5	73.1	50.5	60.4	30	57
YOLO-V5s6u [60]	—	—	—	—	—	—	56.4	—	—
YOLO-V8s [60]	—	—	—	—	—	—	53	—	—
[61]	75.2	**70.1**	—	—	—	—	73.4	—	**72.5**
**SimYOLO-V5s_WIOU**	**77.5**	**67**	**87.2**	**30.8**	**76.2**	**99.5**	**73.4**	**39.4**	**68**

**Table 9:** Comparison of vehicle detection performance (speed) of SimYOLO-V5s_WIOU with state-of-the-art on the DAWN dataset.

Model	Pre-Processing (ms)	Inference (ms)	Post-Processing (ms)	FPS
YOLO-V11s [39]	0.6	26.5	11.1	26
YOLO-V5s+CBAM [38]	0.3	8.0	12.9	47
YOLO-V5s+NAM [38]	0.3	8.2	1.4	101
YOLO-V5s+SimAM [38]	**0.2**	**7.1**	**1.1**	**119**
**SimYOLO-V5s_WIOU**	**0.3**	**7.3**	**3.8**	**88**

#### SimYOLO-V5s_WIOU vehicle detection performance on the FC dataset.

**Fig 9:**
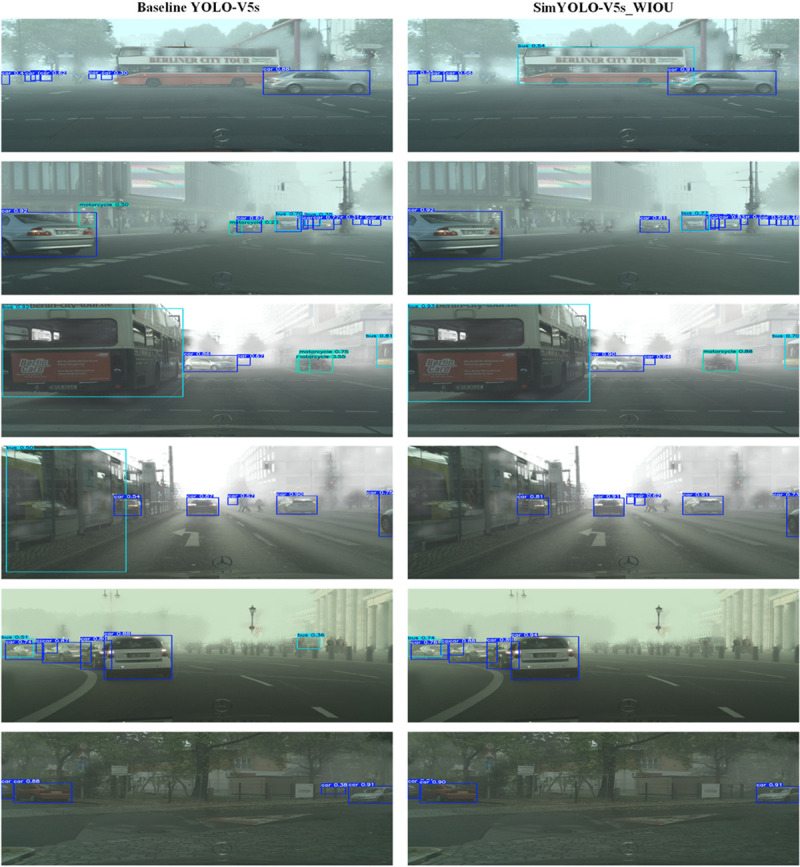
Visual comparison of vehicle detection performance of baseline YOLO-V5s and optimized SimYOLO-V5s_WIOU on set-I.

**Fig 10:**
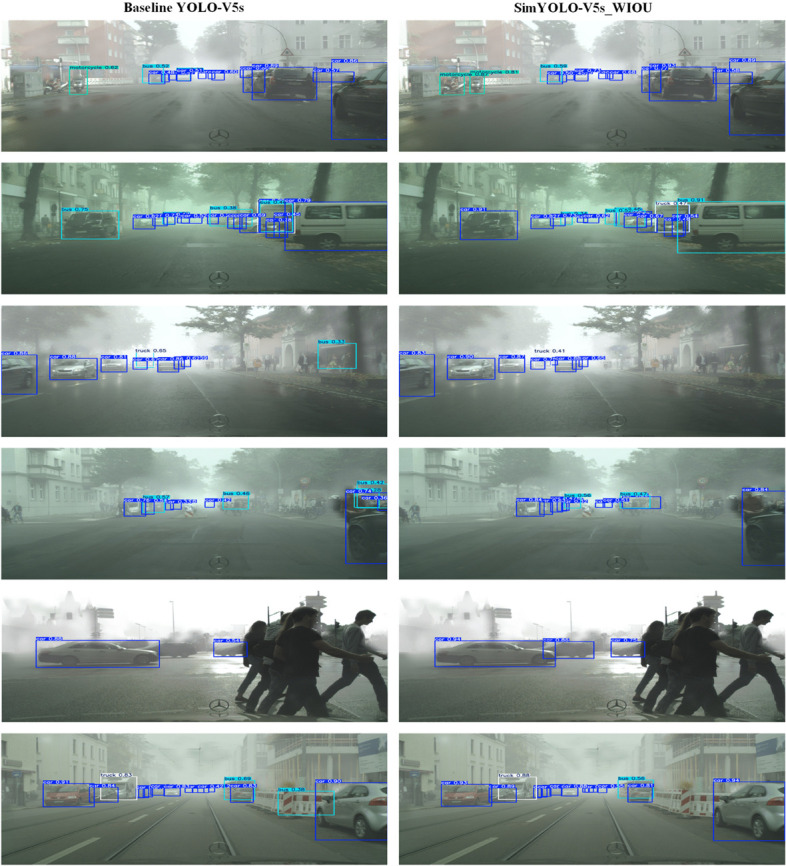
Visual comparison of vehicle detection performance of baseline YOLO-V5s and optimized SimYOLO-V5s_WIOU on set-II.

**Fig 11:**
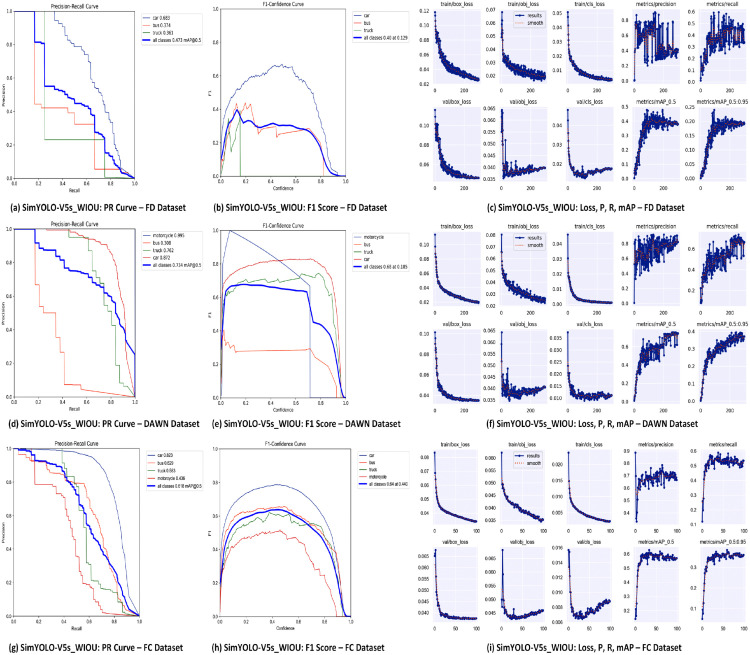
Loss, P, R, and mAP graphs of SimYOLO-V5s_WIOU on FD, DAWN, FC datasets.

The optimized SimYOLO-V5s_WIOU algorithm achieves a multiclass vehicle detection mAP50 score of 61.8%, and a multiclass vehicle detection mAP50-95 score of 38.4% on the FC dataset. The SimYOLO-V5s_WIOU overall precision score is 75.6%, the recall score is 55.5%, and the balanced F1 score is 64%. SimYOLO-V5s_WIOU detects cars with a mAP50 score of 82.3%, buses with a mAP50 score of 62.9%, trucks with a mAP50 score of 58.3%, and motorcycles with a mAP50 score of 43.6%. When compared with the baseline YOLO-V5s algorithm, SimYOLO-V5s_WIOU outperforms in precision, recall, mAP50, mAP50-95, and F1 scores. Moreover, SimYOLO-V5s_WIOU also outperforms in detecting motorcycles in foggy weather, and also shows competitive results in detecting cars, buses, and trucks in foggy weather. SimYOLO-V5s_WIOU outperforms the baseline in terms of pre-processing time and inference time, and offers higher FPS compared to baseline YOLO-V5s. Additionally, SimYOLO-V5s_WIOU requires less training time compared to baseline YOLO-V5s. The comparison of vehicle detection accuracy and speed results on the FC dataset is summarized in Table 4 and Table 5, respectively. The proposed optimized SimYOLO-V5s_WIOU algorithm is also compared with SimYOLO-V5s variants proposed in the previous work on the larger FC dataset. It is evident from the results summarized in Table 10 that SimYOLO-V5s_WIOU outperformed all the previous SimYOLO-V5s variants with a precision score of 75.6%. It also shows competitive results in mAP50, mAP50-95, and F1 scores compared to SimYOLO-V5s variants in detecting vehicles in foggy weather on the FC dataset. Additionally, the proposed SimYOLO-V5s_WIOU algorithm is also compared with SimYOLO-V5s variants in terms of pre-processing, inference, and NMS post-processing time, FPS rate, and training time. The results are summarized in Table 11. The visualization of the vehicle detection performance of baseline YOLO-V5s and optimized SimYOLO-V5s_WIOU is compared and presented in Figs 9 and 10. It is evident from the visualization results of both Set-I Fig 9 and Set-II Fig 10 that the optimized SimYOLO-V5s_WIOU outperforms the baseline YOLO-V5s in correctly detecting vehicle classes, reducing miss and false detections, and achieving higher mAP50 scores. Objectness, classification, and localization loss functions, precision, recall, mAP50, and mAP50-95 scores of SimYOLO-V5s_WIOU vehicle detection algorithm are shown in Fig 11.

**Table 10:** Comparison of vehicle detection performance (accuracy) of SimYOLO-V5s_WIOU with state-of-the-art SimYOLO-V5s variants on the FC dataset.

Model	P	R	Car	Bus	Truck	Motorcycle	mAP50	mAP50-95	F1
SimYOLO-V5s_CIOU [25]	73	53.7	**83.1**	61.6	54.7	**49**	**62.1**	37.9	61
SimYOLO-V5s_DIOU [25]	74.6	53.3	81.7	**67.1**	55.6	37.5	60.5	37.6	61
SimYOLO-V5s_EIOU [25]	67.4	**57.5**	82	**63.9**	55.5	**43.8**	61.3	37.7	62
SimYOLO-V5s_GIOU [25]	67.9	**59.9**	**82.9**	**65.8**	58.1	**48.1**	**63.7**	**38.8**	63
SimYOLO-V5s_SIOU [25]	73.2	**57.8**	81.5	**65.3**	**60.4**	**45.5**	**63.2**	37.8	**65**
**SimYOLO-V5s_WIOU**	**75.6**	55.5	82.3	62.9	58.3	43.6	61.8	38.4	64

**Table 11:** Comparison of vehicle detection performance (speed) of SimYOLO-V5s_WIOU with state-of-the-art SimYOLO-V5s variants on the FC dataset.

Model	GPU-MU	Pre-Processing	Inference	Post-Processing	FPS	TT
SimYOLO-V5s_CIOU [25]	4.01	1.5	45.3	**2.1**	20	5.140
SimYOLO-V5s_DIOU [25]	4.01	1.6	47.6	**2.1**	19	5.074
SimYOLO-V5s_EIOU [25]	4.01	1.5	46.2	**2.1**	20	5.104
SimYOLO-V5s_GIOU [25]	4.01	**1.4**	43.4	**2.1**	21	5.119
SimYOLO-V5s_SIOU [25]	4.01	1.5	46.2	2.4	20	**4.843**
**SimYOLO-V5s_WIOU**	**3.77**	**1.7**	**43.3**	**2.2**	**21**	**4.916**
GPU-MU: GPU Memory Utilization (GB); Pre-Processing, Inference, and Post-Processing (ms); TT: Training Time (Hrs)						

### ECE-VDTDA system: Vehicle tracking

**Fig 12:**
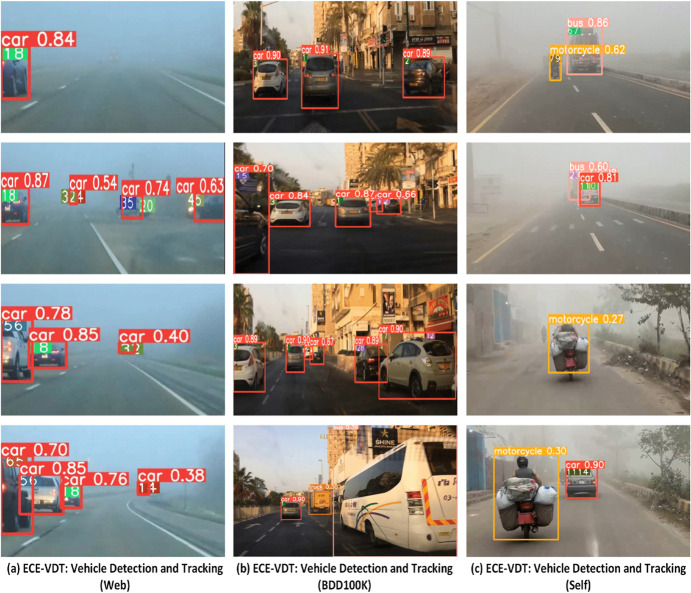
Visualization of Vehicle detection and tracking performance of ECE-VDTDA system.

The vehicle tracking performance of the ECE-VDTDA system is evaluated on diverse video datasets, including BDD100K, web-collected, and self-collected. State-of-the-art Deep-SORT, Strong-SORT, and optimized Deep-SORT algorithms are utilized for the vehicle tracking task, empowered by the optimized SimYOLO-V5s_WIOU vehicle detection algorithm. The comparison of the ECE-VDT performance on the BDD100K dataset video sequence of 1213 frames is summarized in Table 12. It is evident from the comparative results that optimized SimYOLO-V5s_WIOU, along with the optimized Deep-SORT algorithm, outperformed baseline YOLO-V5s, along with baseline Deep-SORT and optimized Deep-SORT algorithms combination in terms of pre-processing time of 0.5 ms, inference time of 11 ms, and post-processing time of 1.6 ms. However, the optimized SimYOLO-V5s_WIOU and the optimized Deep-SORT algorithm combination show competitive results compared to other SimYOLO-V5s variants and the optimized Deep-SORT combination. The comparison of the ECE-VDT performance on the BDD100K dataset video sequence of 1213 frames is summarized in Table 13. It is evident from the results that the optimized SimYOLO-V5s_WIOU and Strong-SORT combination outperformed baseline YOLO-V5s and Strong-SORT in terms of pre-processing time of 0.4 ms, inference time of 14.8 ms, and post-processing time of 1.7 ms. However, the optimized SimYOLO-V5s_WIOU and the Strong-SORT algorithm combination show competitive results compared to other SimYOLO-V5s variants and the Strong-SORT combination. The robustness of the optimized SimYOLO-V5s_WIOU vehicle detection algorithm with baseline Deep-SORT, optimized Deep-SORT, and Strong-SORT vehicle tracking algorithms is further evaluated on a self-collected foggy weather video sequence of 10213 frames. The comparative results are summarized in Table 14. The results indicate that the optimized SimYOLO-V5s_WIOU vehicle detection algorithm enhances the performance of vehicle tracking algorithms, particularly at high speeds. It is also observed that for the vehicle tracking performance of the baseline Deep-SORT, optimized Deep-SORT, and Strong-SORT, the Deep-SORT algorithm switches track IDs more frequently compared to Strong-SORT. However, the processing time and track update time of Strong-SORT are comparatively higher than those of Deep-SORT. The visualization of vehicle detection and tracking performance of the ECE-VDT module on diverse BDD100K, web-collected, and self-collected video datasets of the ECE-VDTDA system is shown in Fig 12.

**Table 12:** Comparison of vehicle detection and tracking performance of the ECE-VDT system with SOTA on the BDD100K video sequence of 1213 frames.

Paper	Detection	Tracking	Pre-Processing	Inference	Post-Processing
[25]	Baseline YOLO-V5s	Baseline Deep-SORT	0.5	11.5	1.9
[25]	Baseline YOLO-V5s	Strong-SORT	0.5	15.9	2.2
[25]	SimYOLO-V5s_CIOU	Optimized Deep-SORT	0.4	11.2	2.0
[25]	SimYOLO-V5s_DIOU	Optimized Deep-SORT	0.4	11.1	2.1
[25]	SimYOLO-V5s_EIOU	Optimized Deep-SORT	0.5	11.5	1.8
[25]	SimYOLO-V5s_GIOU	Optimized Deep-SORT	**0.3**	**10.2**	1.6
[25]	SimYOLO-V5s_SIOU	Optimized Deep-SORT	0.5	11.1	2.3
**Our**	**SimYOLO-V5s_WIOU**	Optimized Deep-SORT	0.5	11	**1.6**
Pre-Processing, Inference, and Post-Processing (ms)					

**Table 13:** Comparison of vehicle detection and tracking performance of the ECE-VDT system with SOTA on the BDD100K video sequence of 1213 frames.

Paper	Detection	Tracking	Pre-Processing	Inference	Post-Processing	Update	Total
[25]	Baseline YOLO-V5s	Strong-SORT	0.5	15.9	2.2	29.1	47.7
[25]	SimYOLO-V5s_CIOU	Strong-SORT	0.5	16.7	1.8	**24.3**	43.3
[25]	SimYOLO-V5s_DIOU	Strong-SORT	0.6	16.0	1.9	31.8	50.3
[25]	SimYOLO-V5s_EIOU	Strong-SORT	0.6	**14.0**	1.7	28.2	44.5
[25]	SimYOLO-V5s_GIOU	Strong-SORT	0.6	15.5	1.7	29.1	46.9
[25]	SimYOLO-V5s_SIOU	Strong-SORT	0.4	16.1	1.9	29.3	47.7
**Our**	**SimYOLO-V5s_WIOU**	Strong-SORT	**0.4**	14.8	**1.7**	25.6	**42.5**
Pre-Processing, Inference, Post-Processing, Update, and Total (ms)							

**Table 14:** Comparison of vehicle detection and tracking performance of the ECE-VDT system with SOTA on the self video sequence of 10213 frames.

Detection	Tracking	Pre-Processing	Inference	Post-Processing
Baseline YOLO-V5s	Baseline Deep-SORT	0.5	**12.3**	**2**
**SimYOLO-V5s_WIOU**	Baseline Deep-SORT	**0.5**	12.8	2.2
Baseline YOLO-V5s	Optimized Deep-SORT	0.9	11.5	1.9
**SimYOLO-V5s_WIOU**	Optimized Deep-SORT	**0.4**	**11.4**	**1.9**
Baseline YOLO-V5s	Strong-SORT	0.7	18.1	2
**SimYOLO-V5s_WIOU**	Strong-SORT	**0.3**	**11.4**	**1.5**
Pre-Processing, Inference, and Post-Processing (ms)				

### ECE-VDTDA system: Driver assistance and collision avoidance

The efficient and cost-effective vehicle detection and tracking performance of the ECE-VDT module enables the driver assistance and collision avoidance functionality of the proposed ECE-VDTDA system. In proposed ECE-VDTDA system, a detection is counted as a True Positive (TP) when SimYOLO-V5s_WIOU correctly localized a vehicle with an IOU ≥ 0.5 and optimized Deep-SORT consistently associated it with the correct track ID. These verified detections are then used for distance, speed, and TTC calculations. A False Positive (FP) occurred when SimYOLO-V5s_WIOU detected a vehicle where none existed, when the IOU with the ground truth was < 0.5, or when optimized Deep-SORT produced an incorrect association (identity mismatch). FP cases were excluded from TTC computations and used only for evaluating Precision, Recall, and mAP. The distance, speed, and TTC thresholds and estimations help to generate collision alerts.

**Fig 13:**
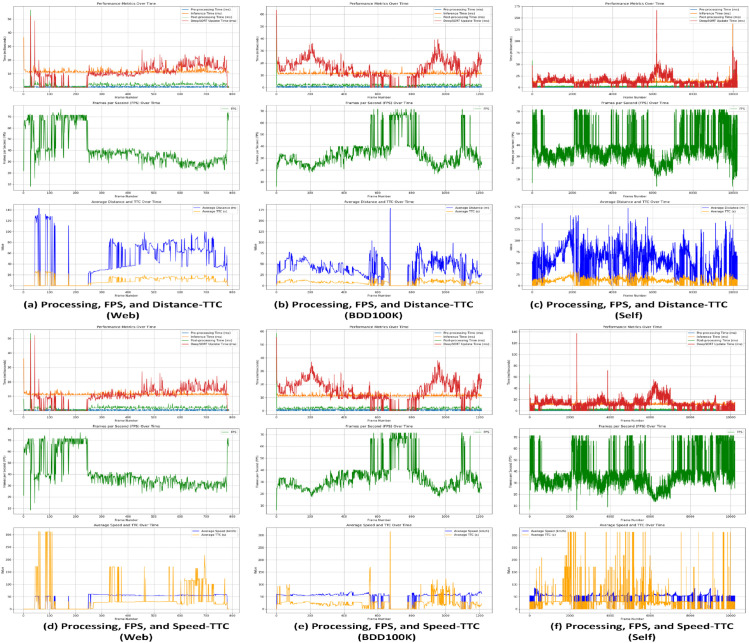
Comparative analysis of processing time, FPS, distance(m)-TTC(s), and speed(km/h)-TTC(s) alerts on web, BDD100K, and self-collected diverse weather datasets for collision avoidance and driver assistance.

**Fig 14:**
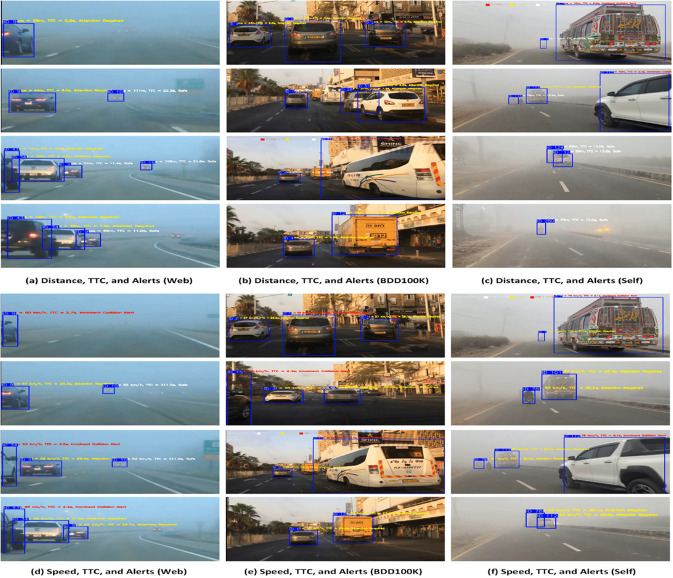
Visual comparison of distance(m)-TTC(s) and speed(km/h)-TTC estimations and alerts on web, BDD100K, and self-collected diverse weather datasets for collision avoidance and driver assistance.

These estimations are an integral part of collision avoidance and driver assistance in the ECE-VDTDA system. Experiments are performed on the diverse web, BDD100K, and self-collected video datasets are utilized for distance, speed, and TTC estimations. The distance-only, speed-only, distance-TTC, and speed-TTC alerts, along with vehicle track IDs, are displayed on the vehicles’ bounding boxes. The process of distance, speed, and TTC estimation is already discussed in the research methodology section. The ECE-VDTDA system’s performance of distance-TTC and speed-TTC estimation over web, BDD100K, and self-collected video datasets is shown in Fig 13. The Fig 13A-13C show the processing time, FPS, and distance-TTC estimations and alerts over video frames. Whereas, Fig 13D-13F show the processing time, FPS, and speed-TTC estimations and alerts over video frames. The distance-TTC relationship is more consistent throughout the video frames, whereas the speed-TTC relationship is comparatively more fluctuating. The FPS processing speed of the ECE-VDTDA system is also competitive, with a rate above 30 FPS and a maximum FPS rate of 70 or higher. The FPS processing speed demonstrates the effectiveness of the proposed ECE-VDTDA systems in real-world settings. The visualization of the ECE-VDTDA system’s performance is provided in Fig 14. The Fig 14A-14C show the distance-TTC estimations and alerts over video frames. Whereas, Fig 14D-14F show the speed-TTC estimations and alerts over video frames. The alerts are displayed on the vehicles’ bounding boxes in different colors, based on the severity of the potential road collisions. The red color shows the imminent road collision warning/alert. The yellow color indicates a collision alert to gain the driver’s attention, while the white color alerts indicate a safe zone for drivers to drive comfortably without requiring focused attention. The ECE-VDTDA system’s performance sets a strong foundation for further exploring collision avoidance and driver assistance design, implementation, real-world testing, and deployment in the modern world’s human-centric and autonomous vehicles.

## Conclusion, limitations and future works

### Conclusion

Emerging human-centric and autonomous diving applications, such as Forward Collision Warning (FCW) and Rear-end Collision Warning (RCW) for Advanced Driver Assistance Systems (ADAS) and Collision Avoidance Systems (CAS), require efficient and cost-effective solutions. In this research work, a vision-based, robust, and computationally efficient vehicle detection, tracking, distance, speed, and Time-To-Collision (TTC) estimation system, Efficient and Cost-Effective Vehicle Detection and Tracking with Driver Assistance (ECE-VDTDA), is proposed for collision avoidance and driver assistance in foggy weather conditions. The vehicle detection performance of the baseline YOLO-V5s algorithm is enhanced by incorporating the SimSPPF module into the backbone and the Wise Intersection Over Union (WIOU) localization loss function. These design changes are incorporated and optimized. A SimYOLO-V5s_WIOU, an efficient and cost-effective vehicle detection algorithm, is proposed. The performance of SimYOLO-V5s_WIOU is evaluated on diverse FD, DAWN, and FC foggy weather datasets and compared with baseline YOLO-V5s and state-of-the-art methods. The optimized SimYOLO-V5s_WIOU outperformed in detection performance and speed. The vehicle tracking performance of baseline Deep-SORT, optimized Deep-SORT, and Strong-SORT is enhanced and evaluated in combination with baseline YOLO-V5s and SimYOLO-V5s_WIOU vehicle detection algorithms on diverse BDD100K, web-collected, and self-collected foggy weather video datasets. The ECE-VDT module, comprising SimYOLO-V5s_WIOU vehicle detection and an optimized Deep-SORT vehicle tracking algorithm, sets a robust and strong foundation for the collision avoidance and driver assistance module. The collision avoidance and driver assistance module comprises distance, speed, and TTC estimations based on pre-defined thresholds and a collision warning/alert mechanism. Distance and speed estimations are linked with TTC separately, and performance is quantitatively and visually compared on diverse video datasets. In proposed ECE-VDTDA system, a detection is counted as a True Positive (TP) when SimYOLO-V5s_WIOU correctly localized a vehicle with an IOU ≥ 0.5 and optimized Deep-SORT consistently associated it with the correct track ID. These verified detections are then used for distance, speed, and TTC calculations. A False Positive (FP) occurred when SimYOLO-V5s_WIOU detected a vehicle where none existed, when the IOU with the ground truth was < 0.5, or when optimized Deep-SORT produced an incorrect association (identity mismatch). FP cases were excluded from TTC computations and used only for evaluating precision, recall, and mAP. The overall functioning of the proposed ECE-VDTDA system establishes a strong foundation for FCW and RCW applications in ADAS and CAS systems.

### Limitations

Several limitations are highlighted in the design and implementation of the proposed ECE-VDTDA system. Predefined threshold levels, scaling factors, and bounding box heights are the primary settings for vision-based distance, speed, and TTC estimations in this research work. A formal probabilistic crash-risk estimate is not included in this study, as such analysis requires advanced statistical frameworks, such as extreme value theory. However, in real-world settings, such as camera installation, adjustment, angle, Field of View (FOV), and Region of Interest (ROI), among other parameters, may need to be further considered and evaluated for the design and implementation of the proposed ECE-VDTDA system.

### Future works

These highlighted limitations are the primary motivation for further fine-tuning the performance of the ECE-VDTDA system. In future work, actual camera parameters will also be incorporated for distance, speed, and TTC estimations. More quantitative observations and measurements will also be considered in the design of the ECE-VDTDA system.
